# A Symbiotic Brain-Machine Interface through Value-Based Decision Making

**DOI:** 10.1371/journal.pone.0014760

**Published:** 2011-03-14

**Authors:** Babak Mahmoudi, Justin C. Sanchez

**Affiliations:** Department of Biomedical Engineering, University of Miami, Coral Gables, Florida, United States of America; University of Vermont, United States of America

## Abstract

**Background:**

In the development of Brain Machine Interfaces (BMIs), there is a great need to enable users to interact with changing environments during the activities of daily life. It is expected that the number and scope of the learning tasks encountered during interaction with the environment as well as the pattern of brain activity will vary over time. These conditions, in addition to neural reorganization, pose a challenge to decoding neural commands for BMIs. We have developed a new BMI framework in which a computational agent symbiotically decoded users' intended actions by utilizing both motor commands and goal information directly from the brain through a continuous Perception-Action-Reward Cycle (PARC).

**Methodology:**

The control architecture designed was based on Actor-Critic learning, which is a PARC-based reinforcement learning method. Our neurophysiology studies in rat models suggested that Nucleus Accumbens (NAcc) contained a rich representation of goal information in terms of predicting the probability of earning reward and it could be translated into an evaluative feedback for adaptation of the decoder with high precision. Simulated neural control experiments showed that the system was able to maintain high performance in decoding neural motor commands during novel tasks or in the presence of reorganization in the neural input. We then implanted a dual micro-wire array in the primary motor cortex (M1) and the NAcc of rat brain and implemented a full closed-loop system in which robot actions were decoded from the single unit activity in M1 based on an evaluative feedback that was estimated from NAcc.

**Conclusions:**

Our results suggest that adapting the BMI decoder with an evaluative feedback that is directly extracted from the brain is a possible solution to the problem of operating BMIs in changing environments with dynamic neural signals. During closed-loop control, the agent was able to solve a reaching task by capturing the action and reward interdependency in the brain.

## Introduction

The design of Brain-Machine Interfaces (BMI) is intended to establish a direct control and communication channel between the central nervous system and prosthetic devices operating in the user's environment. The ultimate vision for BMIs is to assist users in a wide variety of motor tasks encountered in the activities of daily life [1]. Maintaining performance of the BMI while contending with the complexities of daily life activities is a major challenge in BMI system design. However, there are few solutions that can in a hybrid manner contend with dynamics of neural and environmental conditions, which are necessary for clinical neuroprosthetic systems. Interestingly, unlike current BMIs, natural biological systems have emerged in such a way that they are responsive to complex and changing environments. By engaging a Perception-Action-Reward Cycle (PARC) through an intricate set of sensorimotor processes [2], [3] internal antecedents are expressed through actions and ultimately the outcomes of these actions contribute to shaping future motor behaviors [4], [5]. Underlying a large part of this PARC in goal-directed behavior is valuation, which is the process of computing action-outcome sequences to optimize future decision-making in the context of dynamical conditions [6].

While great progress has been made in BMI design, current approaches for decoding neural activity into behavior completely ignore the major aspects of the PARC by relying heavily only on the primary motor representation of behavior [7]–[9]. For BMIs, the acquisition of goal-directed behaviors is critical for reach to grasp motions, which are highly desirable for paralyzed patients [10]. The most popular methods of neural decoding focus on reconstructing hand trajectories from motor neuron activity using input-output modeling derived from electrical engineering applications [11]. These approaches solve functional regression problems but are devoid of the key components that are known to play a role in goal-directed action selection [6]. In addition, unlike the nervous system, these input-output interfaces are typically static and cannot adapt without an external training signal such as the mean square error (MSE) between the true and predicted behavior. As a result, two main paradigms for providing an external training signal have been designed and they can be categorized as *trajectory-based* [12]–[14] or *goal-based* [15], [16] BMIs. In trajectory-based BMIs, the role of the external training signal is to generate correction signals between a known trajectory and an intended trajectory. While this approach is an efficient means of supervised learning, it is difficult to extract the desired kinematics from paralyzed individuals. The goal-based BMIs on the other hand focus on extracting goal information from the brain and leave the execution of motor movements to an intelligent robot actuator that shares control. The goal-based BMIs are ideal for controlled environments with a discrete set of known goals. However, these may not always be known in new environments. In addition, the user also loses control over the motor aspects of the trajectory. Despite these difficulties, these BMIs have proven to exhibit good performance for the context that they are designed for but an important question remains to be answered here. Are the BMIs designed based on these approaches capable of handling the complexities that arise from the dynamics (both neuronal and behavioral) of new tasks during daily life activities?

Designing next generation intelligent neuroprosthetics that actively can evolve with the user to cooperatively maximize a shared goal could be a solution to the problem of interaction with complex dynamical environments [17], [18]. By putting the user and intelligent neuroprosthesis in a shared PARC which is based on the principles of value-based decision making, the user's intent can be expressed through prosthetic actions and outcomes. That action can be evaluated by the user to promote continuous learning. We call this framework a symbiotic brain-machine interface (S-BMI). However unlike the PARC in biologic systems, the PARC in an S-BMI should be modified to incorporate two different entities; one with biologic and the other with artificial intelligence. In order to link these two entities as the elements of a goal-directed system, a minimal set of prerequisites are required which are considered to be instrumental in the theory of value-based decision making [6].

An important requirement for S-BMI design is that adaptation should lead to *cooperation* between the user and intelligent neuroprosthesis to achieve the user's goals. The user's goal provides a basis for evaluation of the neuroprosthesis function where higher values should be assigned to motor actions that increase the probability of achieving the goal. In order to evaluate motor actions, an *outcome measure* is required to be extracted from the user. In this work, we seek to link motor action and reward expectation of the user as an outcome measure. The encoding of goal-directed, rewarding behavior has been localized to many centers in the brain [19]. The action-reward relationships in the brain [20], [21] are instrumental for the S-BMI design. In this regard, the striatum is a key structure that represents action-specific reward values in cortico-basal ganglia loops [22], [23] and encodes reward expectation of actions [24] during goal-directed behavior. It is suggested that the striatum enhances the association between sensory information and motor response followed by reward [25]. In this process, the Nucleus Accumbens (NAcc), a major component of ventral striatum, is known to modulate reward-seeking behavior by associating reward values to sensory information and selecting actions that lead to reward. Integration of reward perception and motor information in the NAcc has given rise to the idea that NAcc serves as limbic-motor interface [26].

In addition to linking limbic to motor representations in the brain, the striatum has been hypothesized to play a major role in linking the reward feedback to action selection in goal-directed behavior [27]. Learning based upon this evaluative feedback is the key feature of the reinforcement learning that makes it appealing as the computational framework of the S-BMI [28]. Here, we seek to build a BMI decoder that learns how to take action based on goal information supplied by the user in the form of an evaluative feedback derived directly from the brain. Using neurocomputational mechanisms of reinforcement learning between the NAcc and the primary motor cortex, it may be possible to engage the PARC and facilitate performance of BMIs in multiple environments.

Systematic development of the S-BMI is structured in three components. In the first part, we introduce an S-BMI decoding architecture and develop the theory for training it. Second, we focus on the neurophysiologic aspects of value and demonstrate how it can be used in the computational architecture. Finally, we introduce a set of experiments to test the functionality of the S-BMI.

## Methods

### A. BMI Control Architecture

By formulating the BMI control as a decision-making problem, the process of optimization can be built on the theory of Markov Decision Processes (MDP) and automated using the well-known approach of reinforcement learning [29]. In the design of S-BMI, learning through reinforcement is very appropriate because it is inspired by operant conditioning of biological systems where the learner must discover which actions yield the most reward through experience [30]. The approach is built on the concept of valuation and as described above, valuation is the process of how a system assigns importance to actions and behavior outcomes. In the design of S-BMI, we seek systems that compute with action-outcome sequences and assign high value to outcomes that yield desirable rewards. This approach is very different from habitual valuation which does not participate in continual self-analysis [31] which is important in dynamical environments. One of the main computational goals in the methods presented here is to develop real-time techniques for modeling and coupling the valuation between the user and the BMI (to enhance symbiosis) in a variety of tasks.

**Figure 1:**
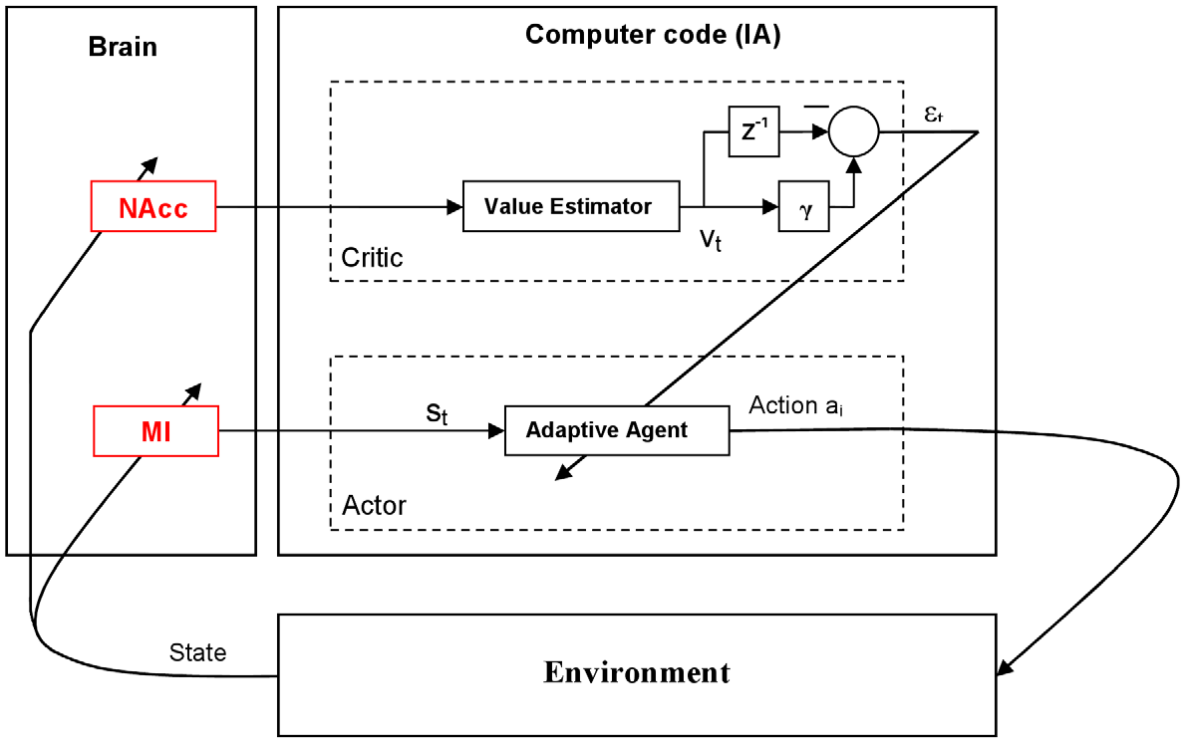
Block diagram of the symbiotic BMI controller. The architecture contains two key components. The Actor is driven by the primary motor cortex (s_t_) and its primary role is to select actions (a_i_) in the environment. These actions are evaluated by the Critic, which is driven by the NAcc. At each instance in time, the Critic provides an error signal (ε_t_) that is computed based on the gradient of reward expectation (v_t_) and is used to adapt the parameters of the Actor for choosing actions that lead to reward. In this entire system, there is in intrinsic coupling between the motor system, reward system, and the environment.

In Figure 1, we formulate the control architecture of the S-BMI based on Actor-Critic implementation of reinforcement learning [32]. In the S-BMI, the Actor plays the rule of decoding the user's neural motor commands. The Actor receives the neural representation of user's intended actions, recorded from the primary motor cortex (M1), as input and translates them into actions in the user's environment. Depending on the internal goals of the user, NAcc represents a reward feedback. The Critic translates this neural feedback into a temporal-difference error (evaluative feedback) for adaptation of the Actor. The architecture in Figure 1 combines key elements of the Actor-Critic framework: actions, states, and value, which are distributed between the user and the computer code which we call an “Intelligent Assistant” (IA).

Note that the evaluation (Critic) and action selection (Actor) subsystems are split into two embodiments (brain and IA), creating a symbiotic brain-machine system due to the real-time feedback. In the Actor-Critic framework, the value of an action is specified by a measure of reward received when that action is selected. At every instance in time, the brain generates new M1 states, the IA selects actions, and the Critic estimates the action outcome based on the representation of reward expectation in NAcc. The update of the state-to-action mapping is based on the past history of rewards and the estimation of future rewards. The modulation of reward activity in the user's brain defines the task, which is a great advantage for reaching tasks in the external world because the designer does not need to specify the reward function in the environment as is done in conventional reinforcement learning paradigms [32]. The agent finds an optimal control strategy based on the user's neuronal state and the actions that are defined as movement direction. The key problems in this architecture are the following:

1. Translate the neural population NAcc activity into a scalar evaluative feedback signal. This involves the integration of improved real-time signal processing methods that capture global computation on multiple spatial, temporal, and behavioral scales.
2. Estimate the state-action value function (shown mathematically later) that selects future actions given the states. To best capture the effects of the neural inputs on the architecture, initialization, and parameter selection of the Actor-Critic model, we will perform our experimentation first in a simulator and then on real data collected from a behaving animal.

#### Critic structure

**Figure 2:**
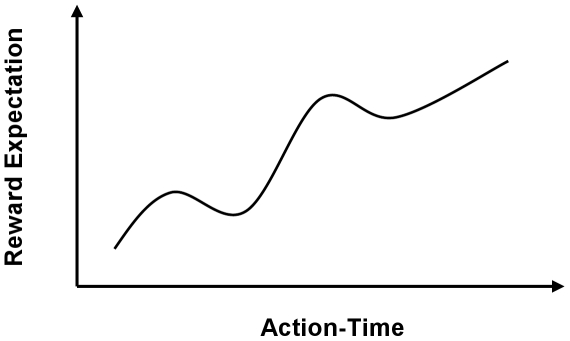
Conceptual diagram of reward expectation modulation of the user based on IA actions. The temporal structure of NAcc neuronal activity indicates the expectation of reward or aversion in goal directed tasks. The Critic must interpret this activity and transform it to a scalar error signal.

To address problem (1) above, the traditional use of the Critic in reinforcement learning must be reformulated. In our architecture, the value function was implemented in the brain and it is biologically trained. Therefore, we just need to estimate the evaluative feedback from the neural population response in NAcc. Depending upon the user's goal, IA actions might increase or decrease the reward expectation [33]. During BMI use, we sought to best capture and model the response of NAcc neurons over time and translate it into a scalar value that could be used for evaluation of actions of the Actor. We modeled the hidden parameters in the NAcc data that pertained to goal proximity and movement directions. By finding the modulatory effect of IA actions on the reward expectation of the user, the value function predicted how the actions over time influenced the reward expectation. Figure 2 schematically plots the reward expectation as a function of IA's actions over time where the positive slope corresponds to approaching the goal and negative slope represents getting away from the goal. Training the Critic involved translating the NAcc neural activity to a scalar function that predicted the gradient of the reward expectation of the user in presence of known goals. A Time-Delayed Neural Network was trained using conventional error backpropagation [34] for this purpose. The network was composed of tap-delayed lines at the input to capture the temporal structure of the NAcc activity and a Multi-Layer Perceptron with a linear output Processing Element for estimating a scalar evaluative feedback from the multi-channel NAcc neural activity.

#### Actor structure

The Actor was a parameterized policy estimator that treated neural activity in M1 as a Markov process. The role of the actor is to find a mapping between user's neural states and robot actions to maximize a measure of the user's reward expectation that was presented by the Critic. The user's reward expectation (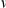) was a function of the IA's actions and user's neural state. 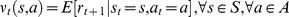
(1)At each time step, the Actor, which was parameterized by 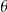
_,_ took action under policy π. 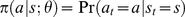
(2)The cost function was defined as the average of expected reward over time.
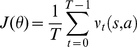
(3)


The Actor should find a set of 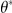
that maximized 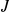 over time, i.e. 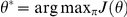. As the Actor converged to the optimal policy, the user can actively control the actions by modulating appropriate neural states. During adaptation, the Actor estimated the gradient of 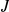 with respect to states and actions and improved the policy by adjusting its parameters 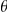 in the direction of 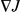 therefore an instantaneous measure of gradient direction was required. We defined an instantaneous error that resembled the temporal difference error in the regular Actor-Critic architecture as an estimate of 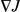


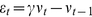
(4)


To approximate the optimal policy, we used a Time-Delayed Neural Network. As in other BMI experiments, we use the firing rate as the input over all channels within 100 msec windows, which have been embedded in longer time windows using a gamma memory structure [35]. The network architecture was composed of a set of nodes that received the M1 neural state as input. A hidden layer formed a set of basis functions and finally the output layer, which spanned an action space for IA. The exact specification of the number of processing elements is presented in the results section. In a discrete action space, each output processing element represented one action and computed the value of the corresponding action given parameter set 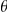 and input neural state *S_t_* (Eq. 2). The Actor executed the action with the highest value and received an evaluative feedback from the Critic. The evaluative feedback was computed by Eq. 4 and backpropagated [34] to the Actor network to adjust the parameters of the selected action (Eq. 5).
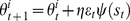
(5)Here 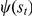 represented the projection of input M1 neural state to a feature space spanned by the hidden layer of the multi-layer perceptron in the Actor structure. The superscript in Eq. 5 corresponds to the index of selected action. Table 1 summarizes the Actor's adaptation procedure in the S-BMI architecture.

**Table 1:** Adaptation algorithm of the Actor structure.

1	The user generates motor state s_t_ and its reward expectation is 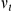 .
2	The Actor associates s_t_ to action a_i_ and executes the selected action.
3	Execution of action a_i_ increases or decreases the reward expectation, which would be reflected by 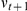 .
4	The error is defined as 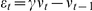
5	If 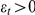
	This error is used to update the parameters of selected action.
	The hidden layer weights are not changed.
	If 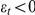
	Parameters of selected action would be updated.
	The error back propagates to the hidden layer and updates the hidden layer weights.
6	Return to step one.

### B. Neurophysiology

**Figure 3:**
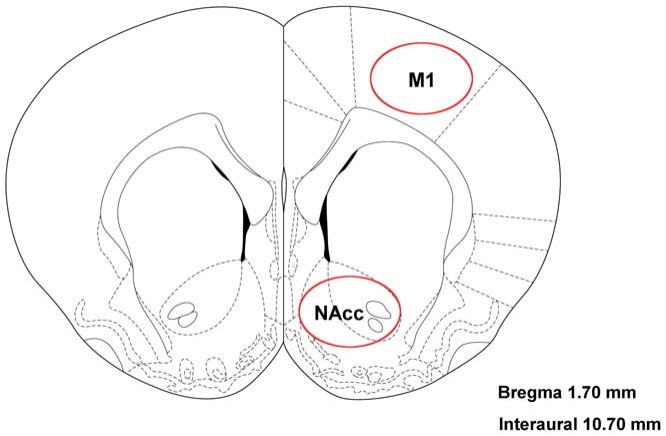
Stereotaxic neurosurgical methods were used to target the NAcc and M1. In experiments involving simultaneous recording of NAcc and M1, a dual electrode array was implanted.

**Figure 4:**
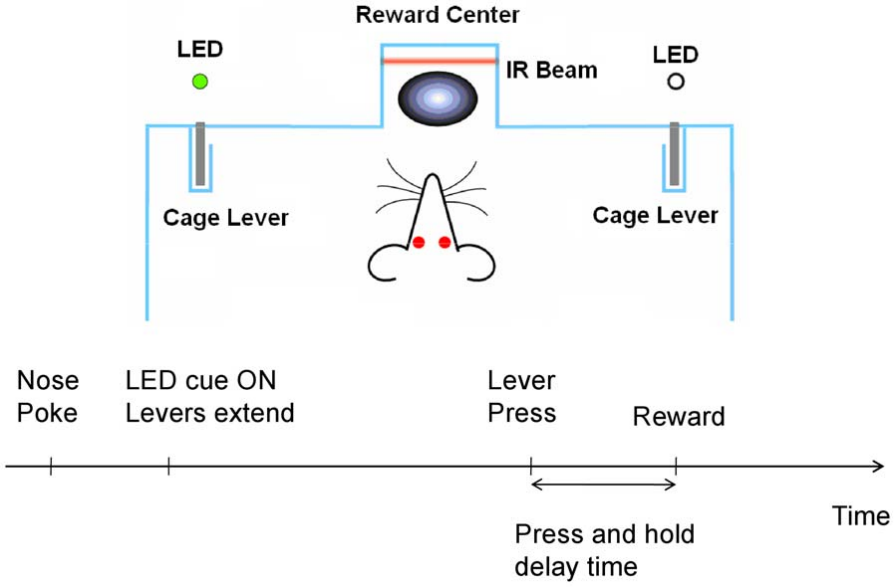
Top view of the animal behavioral box. A nose poke into the IR beam initiated the random selection of a target level cued by a light (LED). The animal had up to 4 seconds to press a lever. If the correct lever was pressed, a water reward was delivered.

Since the Actor-Critic architecture depended heavily on the evaluative feedback from the NAcc, we first performed neurophysiological studies to characterize the evaluative feedback information in it and its appropriateness as a training signal for the S-BMI architecture. We implanted microwire array electrodes into the left NAcc of three Sprague-Dawley rats and chronically recorded single unit activity of accumbal neurons during a reaching task. Each array was 8×2 electrodes (16 total) with 250 µm row and 500 µm column spacing (Tucker-Davis Technologies, Alachua FL). The arrays were positioned stereotaxically and lowered with a hydraulic micro-positioner to an approximate depth of 7.0±0.2 mm (Figure 3) [36]. This site was chosen because of the high density of medium spiny neurons at this level [37]. Additional details of the surgical technique are given in [38]. The rats were given up to two weeks to recover from surgery before resuming the experiment. All procedures were approved by the university Institutional Animal Care and Use Committee (IACUC). All rats were trained in a two-lever choice task via operant conditioning to earn water reward by pressing retractable levers (Med Associates, St. Albans VT) inside their behavioral chamber cued by lights (LEDs) (Figure 4). A solenoid controller (Med Associates) dispensed 0.04 mL of water into the *reward center* on successful trials. The press and hold time was variable between 0.125 to 0.5 seconds. An IR beam (Med Associates) passed through the most distal portion of the reward center. The workspace used low-level lighting and was designed to maximize the rat's visual abilities. After the rats reached the operant conditioning inclusion criteria of 80% on each side, neural data was recorded for six sessions.

Electrophysiological recordings were performed using commercial neural recording hardware (Tucker-Davis Technologies, Alachua FL). A system (one RX5 and two RP2 modules) operated synchronously at 24414.06 Hz to record neuronal potentials from microelectrode arrays. The neuronal potentials were band-pass filtered (0.5–6 kHz). Next, online spike sorting [39] was performed to isolate single neurons in the vicinity of each electrode. Prior to the *in vivo* recording, the experimenter reviewed each sorted unit over multiple days to refine the spike sorting thresholds and templates. The number of sorted single units varied between rats: rat01 had 12 units, rat02 had 13 units (including one multi-unit), and rat03 had 41 units. The isolation of these units was repeatable over sessions with high confidence from the recordings. Once the neurons were isolated, the data acquisition system recorded unit firing times and a firing rate estimate was obtained by summing firing within non-overlapping 100 ms bins. Additionally, all behavioral signals (e.g. water rewards, light activation) were recorded synchronously using the shared time clock.

### C. Closed-loop Simulator

The first step in systematically testing the functionality of the Actor-Critic architecture driven by both M1 and NAcc was to develop a simulator. The simulator offers the possibility of presenting environmental changes and inputs with known characteristics that allows the study of how both interact with the model initial conditions, parameter selection, convergence and overall performance. Since it was difficult to control every aspect of neuronal responses during *in vivo* experiments, the simulator offered a method to investigate these aspects before running closed-loop experiments with the animal in the loop. The simulator was composed of three main modules: neural firing synthesizer, Actor-Critic controller, and environment. The environment could be specified as 2D or 3D. However, for simplicity we first began with a 2D environment that consisted of a 20×20 grid world with 0.1 spacing between each node. The task was to navigate a robotic arm from the center of the grid to any target in 2D space based on the neural representation of the motor commands and a scalar evaluative feedback. The neural firing synthesizer in our simulation consisted of an ensemble of synthetic M1 neurons that were generated based on the model presented in [40]. The main parameter of neural firing module was the tuning properties of the neurons. The ensemble of cortical neurons was composed of four subsets where neurons in each subset were tuned to a principal direction (up, down, right, and left) in the workspace. At each time step, the neural firing synthesizer produced a motor command that was encoded into M1 neural activity by exciting the corresponding subsets of neurons. For example, if the user decided to navigate the robot in the up-right direction, those neurons in the ensemble which were tuned to the right direction and up direction were activated. The Actor-Critic controller received neural input from the neural firing simulator and used it to navigate. Next, we required a synthesizer of the NAcc evaluative feedback. To approximate both rewarding and aversive evaluative feedback we used the cosine of the angle between the robotic arm movement and the direct path to the target. With this simulation, behaviors that are maximally rewarding are directly in the direction of the target and would yield the cosine of zero, which is a maximally positive value. Conversely if the movement was in the opposite direction of the target the behavior would be aversive and maximally negative. Based on the robot movement with respect to the target, movement and target vectors were computed at each time step. We selected cosine function because it converted movements towards target (−90<θ<90) into a positive and movements away from the target (90<θ<270) to a negative value. This error signal resembled the temporal-difference error δ in the Actor-Critic algorithm. Each experiment was composed of 100 trials where each trial consisted of a single reach to a specified target. In each trial, if the agent could not reach the target in 50 steps the trial was considered unsuccessful.

### D. Simultaneous Decoding using M1 and NAcc

After the simulator analysis was completed, a real S-BMI experiment was conducted to test the Actor-Critic decoding performance using real M1 and NAcc neural activity. Here a 3-D reaching task was completed by modifying the experiment setup in section II.B by adding a robotic arm as in [28]. The robot workspace was in front of the rat's cage and the rat was able to see it through Plexiglas cage wall. Two levers on the left and right sides of the robot workspace were used as targets. The distal target was cued by light (LED) and the task was to navigate the robot to the distal target. Once the robot reached the target, a water reward was delivered to the rat. A Male Sprague-Dawley rat was trained in a two-lever choice task via operant conditioning to associate robot control with earning water reward [41]. The behavioral procedure was similar to those in II.B. except that instead of the rat manually pressed the levers inside the cage to obtain water reward, a robotic arm was used to press the set of distal levers. The training paradigm was designed to shift the attention of the rat to the movements of the robot. Once the rat made association between the reward and robot actions, catch trials were introduced in which the robot moved to the lever that was not cued (non-target lever). In this case the rat received an aversive feedback (negative tone with no water reward). These catch trials provide contrast between rewarding and aversive target and allow more detailed study of the evaluative feedback signal used to train the S-BMI. The order of target and non-target trials was random throughout the training session but they were balanced to keep the rat motivated.

After reaching an 80% operant conditioning inclusion criterion, the rat was chronically implanted unilaterally with a custom designed microelectrode array (32 electrodes) that simultaneously targeted the layer V of the forelimb area in the M1 [42], [43] and the NAcc (see Figure 3). Each array was 8×2 electrodes with 250 µm row and 500 µm column spacing (Tucker Davis Technologies (TDT), Alachua FL) but the length of arrays for MI and NAcc were different to target each structure. The arrays were positioned stereotaxically and lowered simultaneously with a hydraulic micro-positioner to an approximate depth of 1.6 mm for the MI array and 7.5 mm for the NAcc array. Spatio-temporal characteristics of neuronal signal during insertion provided additional information about the array location relative to neurophysiologic landmarks. The rat was given up to two weeks to recover from surgery before resuming the experiment. Prior to the first closed-loop experiment, the experimenter reviewed each sorted unit over multiple days to refine the spike sorting thresholds and templates. In this experiment, 20 single units in M1 and 23 single units in NAcc were isolated. The isolation of these units was repeatable over sessions with high confidence from the recordings. The simultaneous M1 and NAcc recordings were used to derive the Actor-Critic architecture in Figure 1. Testing of the decoding performance consisted of adapting a naïve Actor (randomized weights) using the output of the Critic.

## Results

The development of the Actor-Critic architecture for S-BMI required testing and validation of three characteristics of its design. Innovation in the Actor-Critic design is rooted in the new training signal from NAcc, the ability to adapt to environmental changes, and the ability to respond to neural plasticity. Therefore we quantified the properties and performance in the following areas.

- Temporal properties of the evaluative feedback signal from NAcc which will be used to train the network
- Convergence properties of the Actor-Critic during environmental changes
- The effect of neural reorganization on Actor-Critic generalization
- Simultaneous use of real M1 and NAcc activity in decoding

The results section is composed of four parts. First, we performed a neurophysiology study of NAcc during a reaching task and quantify and model the temporal structure of neural ensemble firing leading up to reward. This defined the expected nature of the temporal-difference error signal. Second, we tested how changes in sequential novel target locations affect the convergence of the network. Third, we introduced neural plasticity at the input to the Actor-Critic model and tested the performance over time. Fourth, we used real M1 and NAcc activity to test decoding performance of the full system.

### A. Temporal Properties of NAcc Activity Leading up to Reward

**Figure 5:**
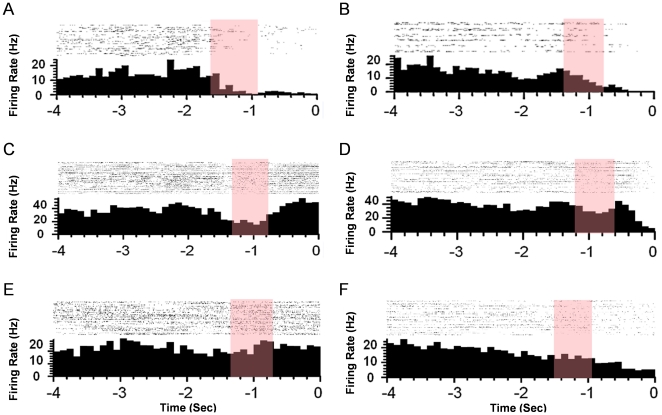
Perievent time histogram of 3 representative NAcc neurons. Dual-nonselective neurons (both decrease firing after cue) for (A) left and (B) right trials. Dual-selective neurons (increase and decrease firing for both targets) (C) left and (D) right trials, and uni-selective neurons (decrease for one and stay constant for the other target) for (E) left and (F) right trials.

Since a biological, neural-based error signal was used to adapt the network using reinforcement learning, a set of guidelines for what can be expected from the temporal modulation of the NAcc leading up to target acquisition was developed here. We are interested in the segment of time between target selection and acquisition and measure how accumbal neurons are excited or inhibited leading up to the target providing reward. To investigate this aspect, the data from the rat lever pressing experiments was segmented so that each trial was time aligned to the onset of the lever press indicated by time 0 in Figure 5 (panels A–F). Next, 4 seconds of data leading up to this point was extracted which we will call the “target acquisition time”. This duration was selected because it corresponded to the maximum time between cue and press for all animals and trials. During the target acquisition time, neuronal firing was binned into 100ms windows and a firing rate was computed. The perievent time histograms in Figure 5 (panels A–F) correspond to the average firing rate over left or right trials during the target acquisition time. Also included are the individual raster plots for each trial.

In this analysis, three groups emerged in the neuronal firing and representative plots of the firing are presented. We performed statistical analysis to identify neurons in each group quantitatively. For each neuron we compared the baseline activity (2 seconds before the cue as indicated by the red bar) with the neural activity during 2 seconds before the lever press using Kolmogorov-Smirnov test (KS-test). The first group of neurons responded non-selectively to both targets. As the animal approached to each target, the neurons in the first group increased or decreased their firing rates. In the second group, neurons selectively responded to each target. The neurons in this category exhibited excitatory and inhibitory activity when the animal approached the left and right targets respectively. The neurons in the third group responded only when the rat approached either target and they did not respond to the other target. Out of 66 isolated neurons (63%) significantly changed their firing rate during goal-approach behavior compared to their baseline activity. In accordance with the three categories that we identified, (46%) belonged to the first category, (25%) belonged to the second category and (39%) belonged to the third category.

The result of our neurophysiology study suggested that there is a hetereogeneous and rich representation of goal information in the NAcc during goal-approach behavior. The next step in the design of the Actor-Critic was to transform this neural representation into a scalar evaluative feedback error signal for adaptation of the Actor. As in the conventional Actor-Critic learning, both the negative and positive reinforcement are required for training the Actor. Extracting the positive and the negative components of evaluative feedback is supported by both our observations here and in the literature with respect to representation of reward and aversion prediction [44].

### B. Convergence of the Actor-Critic during environmental changes

One of the primary advantages of the Actor-Critic architecture is that it is designed to symbiotically adapt with the user during environmental changes. The goal here is to perform simulated closed-loop experiments will be to determine how the Actor-Critic model performance is affected when a new target (unforeseen to the user) is introduced to the behavioral workspace. The addition of new targets is a common occurrence in the activities of daily life and it is expected that with each new target there are unseen aspects of the control scheme that need to be learned and the process will take time. Because the Actor-Critic learns on-line and can respond to changes (unlike static BMIs which learn from a training set), we will test the condition where the navigation of robot to a new target will require the selection of a new action (or action set) to be learned to reach the new target. The new target will be located outside the space spanned by the previously leaned control policy therefore the IA will not be able to reach the new target without the learning to acquire this new action set. The experimental approach was designed to introduce a perturbation to the BMI control paradigm so that the performance difference could be measured. In this section, we specifically focus on an important question regarding the applicability of BMI in daily life activities. How does learning a new task affect the previously learned functional mapping for BMI control?

For these experiments, the task was to navigate to a set of targets located at each of the corners of a square 2D workspace. However, all of the targets are not presented at the same time. Targets were numbered as following: 1-Upper-right, 2-Lower-left, 3-Upper-left and 4-Lower-right. Starting from a naïve state (random small initial Actor weight values between −0.5 and 0.5), the Actor-Critic decoder was required to adaptively find a control policy to reach each target using only the synthesized M1 and NAcc activity. Once the decoder found an appropriate control policy for the task, a new target was introduced. In this way, we presented all the four targets sequentially (1–4) therefore the decoder had to change its control policy for reaching each target. For the first target, the parameters of the decoder were initialized randomly but afterward the network started from the previously learned control policy (i.e. previous Actor weight values). Once the Actor-Critic learned each task individually, we presented all the four targets where, one of the four targets was presented randomly in each trial. In this task, the decoder had to derive a control policy that enabled switching among all the targets. In other words the network had to remember its previous control policies. In each of the tasks, to consolidate the control, as the decoder learned a control policy the learning rate was annealed to zero and parameters of the decoder were frozen. However, at the introduction of a new task, the learning rate was reset and the network resumed adaptation. The learning rate annealing is an important aspect of co-adaptation because it controls to what extent the BMI adapts to the user. In this S-BMI architecture in particular, there were two reasons for annealing the learning rate. First, from machine learning point of view, every time the decoder successfully completes the task the association between the M1 neural states and Actor-Critic actions are reinforced by increasing the corresponding network weights. Annealing the learning rate prevents the network weights from growing unlimitedly. Second, based on the representation of reward, the NAcc become habituated to specific goals over time [45], [46] and may reduce the amount of evaluative feedback over time.

**Figure 6:**
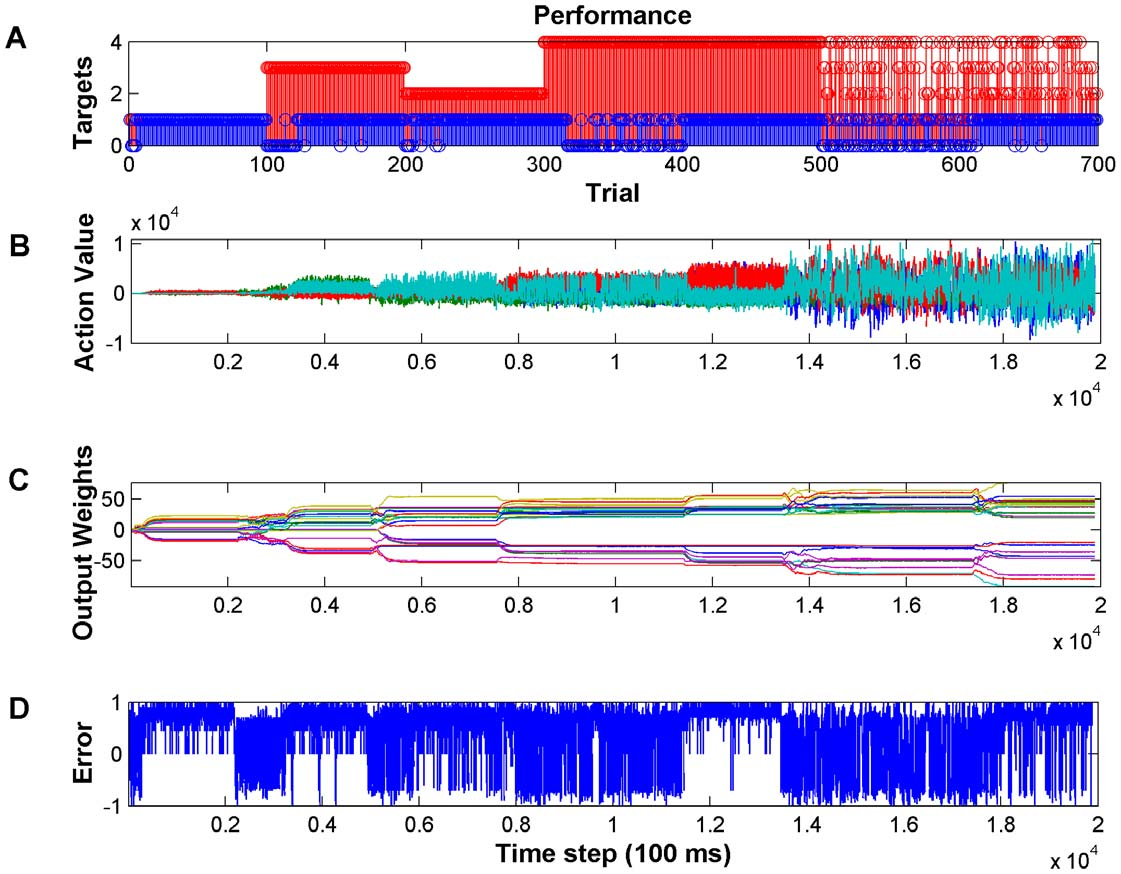
Decoding performance during sequential presentation of the targets in the four-target configuration. (A) Sequential presentation of 4 targets as indicated by red stems. Blue stems indicate if the target was acquired (1) or missed (0). Note that when a new target is introduced the performance decreases but within a few trials it recovers. (B) Temporal sequence of action values. Each colored trace represents the value of one action (i.e. up, down, left, right). Note that for each target only certain actions have high value since they are required to acquire the target. (C) Weight values for the output layer of the Actor. Each colored trace corresponds to an individual weight. Note that when a new target is introduced that the weights adapt then plateau once the performance improves. (D) The temporal difference error becomes maximally positive when the targets are acquired.

Figure 6 shows the performance of the decoder during this set of experiments. The red stem plot in Figure 6A shows the targets that were presented during each trial. The blue stems in Figure 6A show whether the decoder was successful or not in the corresponding trial. Here, we can see by introducing a new target (e.g. at trial 100), performance degraded at the beginning but after a few trials the decoder was able to learn all the new tasks. Table 2 summarizes the decoding performance during sequential target acquisition task. For each target, 100 trials were presented. The number of trials that took for the decoder to find the target during the first 50 trials of each target was used as a measure of speed of learning. The percentage of successful trials during the second 50 trials was used as a measure of performance. If the performance was less than 90%, another epoch of 100 trials was presented to the decoder. Recall, that the output of the Actor network provides the value of each action take. Figure 6B shows the values of the Actor output processing elements over time where actions left, right, up and down are represented by colors blue, green, red and light blue respectively. For each task, we can see the network adjusted its parameters in such a way that maximized the probability of selecting actions that were required for accomplishing the task. For example, in trials 100–200 actions down and right were necessary for reaching the target. These are the actions with the highest value (green, light blue). However, when all targets were presented during trials 500–800 we can see that a mixture of actions had high value and these modulated depending on the target and feedback from NAcc. In Figure 6C we can see how the network adjusted the output layer weights to find a mapping between neural states and optimal actions based on the error signal. It is important to note that at the introduction of a new target, we can observe adaptation in the weight values and then consolidation of the control scheme through the plateau of the weight values. When an environmental change occurred, again the weights adapted appropriately. To guide the adaptation, the Critic provided the reinforcement signal here. It was the reward expectation of the user, which was approximated by the cosine of the angle between movement vector and target vector. From Figure 6D, we can see that when a new target was introduced, the evaluative feedback was the largest in the negative direction (−1 because the movement direction and desired direction are 180 degrees apart). Over time, through adaptation the movement direction and target direction become collinear and the cosine becomes maximally positive. We emphasize here that all of the adaptation of the decoder was through NAcc evaluative feedback and there was no *a priori* training or any external training signal.

**Table 2:** Decoding performance during sequential target acquisition.

	Target 1	Target 2	Target 3	Target 4		All 4 Targets	
Trials	1–100	1–100	1–100	1–100	101–200	1–100	101–200
**Speed** 1	5	8	22	-	6	-	10
**Accuracy** 2	100%	100%	96%	46%	100%	40%	98%

1 Speed is defined by the number of trials to converge to a control policy to solve the task consistently.

2 Accuracy is defined by the rate of success after convergence. For the first 100 trials of “Target 4” acquisition and “All 4 Targets” tasks that the decoder did not converge, the accuracy is computed during adaptation.

### C. Reorganization of neural representation

Brain plasticity is an important design factor of BMI and it has been observed in the context of many research areas [47], [48]. From signal processing point of view, changes in the pattern of neural activity can be a challenging problem for decoding because standard, static input output models assume stationarity in the neural input [49]. In this section, we investigated the reorganization in the neural pattern in an extreme situation where after the decoder converged to a control policy the input pattern for all neurons was perturbed by shuffling the action preferences. During these experiments, the task again was reaching targets that were located at each corner of the workspace (4-target task).

**Figure 7:**
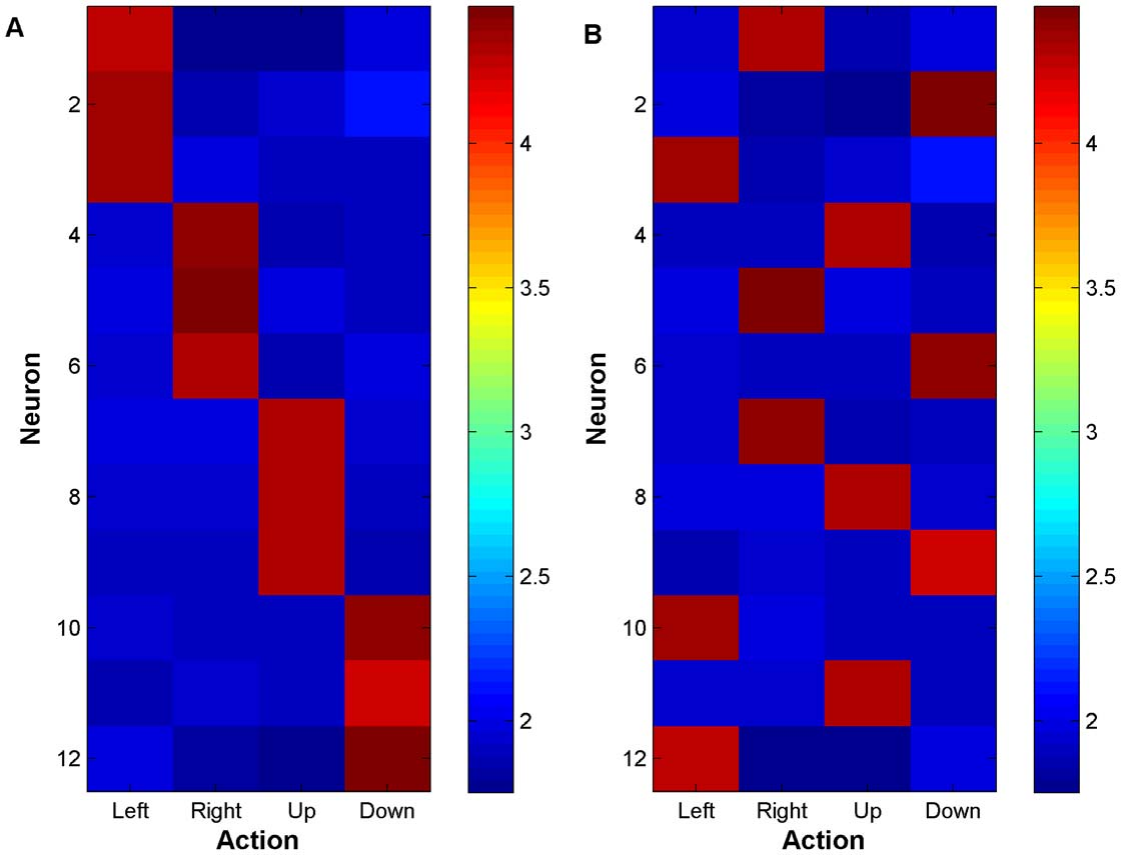
Neural tuning map of the synthetic M1 neurons. (A) before and (B) after reorganization. Here ‘hot’ colors indicate maximal firing.

**Figure 8:**
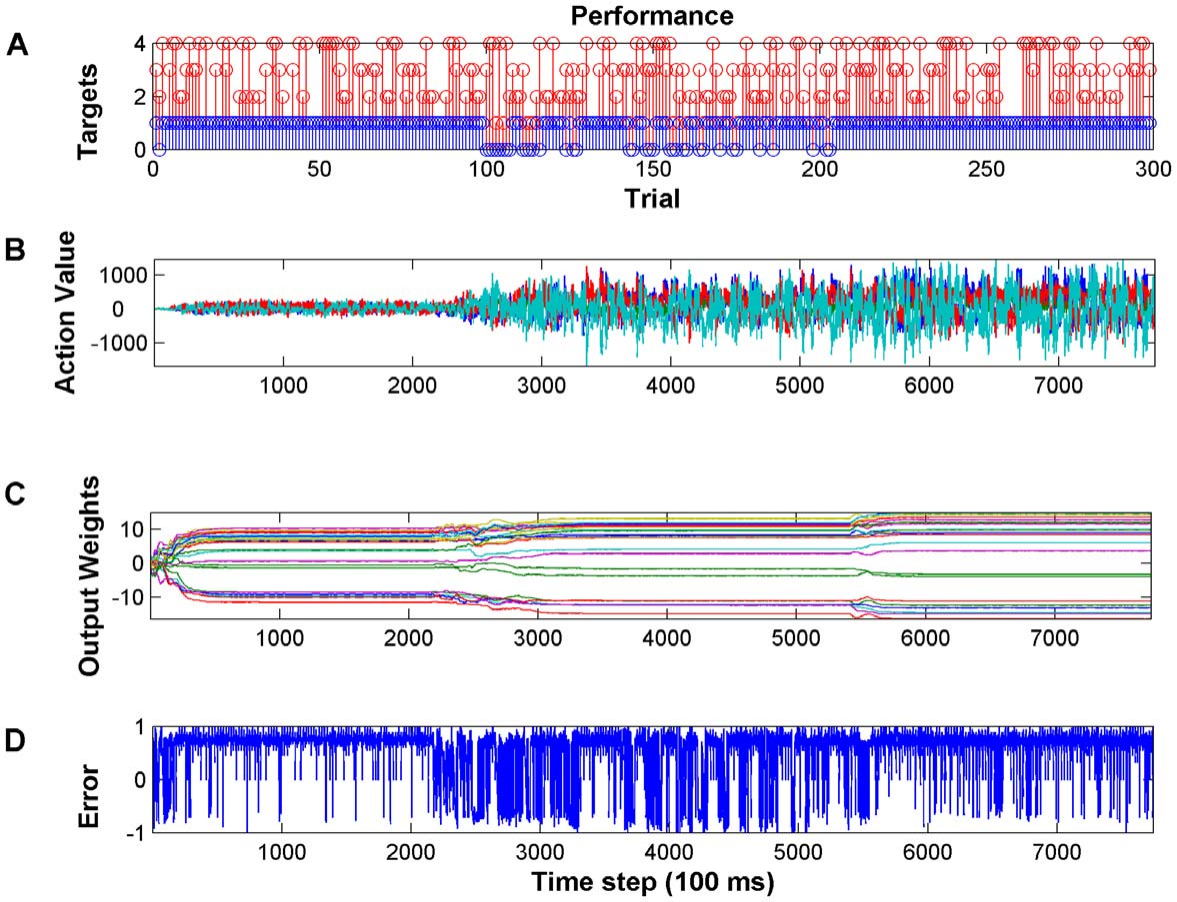
Network adaptation after reorganization of the tuning map. At trial 100, the reorganization was imposed. (A) Performance degradation as indicated by the blue stems marking (0) were observed at trial 100. However, with adaptation perfect performance was regained at trial 200. (B–C) A decrease in performance was matched with adaptation of the action values and weight values to compensate for neural changes. (D) Evaluative feedback in terms of the error here is shown to modulate more frequently when the performance is poor but it stabilizes once performance is regained.

We defined a specific tuning map by dividing 12 neurons in an ensemble into four subgroups each tuned to one of the principal directions that were mentioned in the previous section (see Figure 7A). Here neurons 1–3 were tuned to left, 4–6 right, 7–9 up, and 10–12 down. In this environment, a naïve decoder learned to perform the task perfectly. The first 100 trials of Figure 8 shows the performance of the decoder with this tuning map. For each trial, one of the four targets was picked randomly. We can see the decoder reached 100% accuracy after 3 trials as shown by the convergence of the weights and maximally positive error signal. At this point, the tuning map was perturbed by shuffling the preferred action of the neurons. Figure 7B shows the tuning map of the neural ensemble after reorganization. Here, the tuning is randomly distributed among the 12 neurons.

In Figure 8 (trials 100–300) we can see after reorganizing the neural tuning map, the performance degraded at trial 100 but the decoder was able to recover performance. By letting the decoder adapt more, we can see during trials 200–300 the performance could reach to the level that was before reorganization where the decoder performed the task perfectly.

### D. Robot control using simultaneous M1 and NAcc

Knowledge of NAcc representation and Actor-Critic BMI adaptation were tied together in a full closed-loop experiment with real data. In this section, we used the NAcc neural activity that was recorded simultaneously with M1 to navigate a robotic arm in 3-D space. Since we have learned that goal approach behavior affected the reward expectation and modulation of the NAcc neural activity, here the Critic feedback was defined based on the robot movement trajectory toward the targets. In this way, the Critic was designed as a state estimator. If the robot moved towards the target (Rewarding states) the NAcc neural activity was classified as rewarding and a positive value (+1) was generated; otherwise, if the robot moved away from the target (Aversive states) then the NAcc neural activity was mapped to a negative value (−1). The output of the Critic then was used to adapt the Actor to find mapping between M1 neural activity and robot actions that lead to the target. The robot actions were defined as 12 movement directions in 3D space and the task of the Actor was to select the correct sequence of actions (based on M1 and NAcc neural activity) that navigated the robot to the target. In this setup, the Actor was initialized as completely naïve (small random weight values) and the task was reaching to one target in 3D space.

The same Critic and Actor structures that were introduced in section II.A were used in this experiment. For the Critic, 3 tap-delayed lines were used at the input of the Multi-Layer Perceptron (MLP) network. The MLP was composed of 5 non-linear Processing Elements (PEs) at the hidden layer and 1 linear Processing Element at the output. The output value was thresholded at zero and at each time step during the trial the NAcc neural vector was mapped to +1 or −1. Half of the trials were used for training the Critic and its performance was tested on the other half. The classification performance on the test set was 72% for providing the correct evaluative feedback of +1 or −1. In the test set, the parameters of the Critic were fixed and the output of the Critic was used to train the Actor. The architecture of the Actor was composed of 3 gamma-tap delay lines at the input with an MLP network with 3 non-linear PEs at the hidden layer and 12 linear PEs at the output. At each time step, the Actor received M1 neural vector at the input and computed robot action (movement directions) values at the output. The Critic's response was used to adapt the Actor parameters.

**Figure 9:**
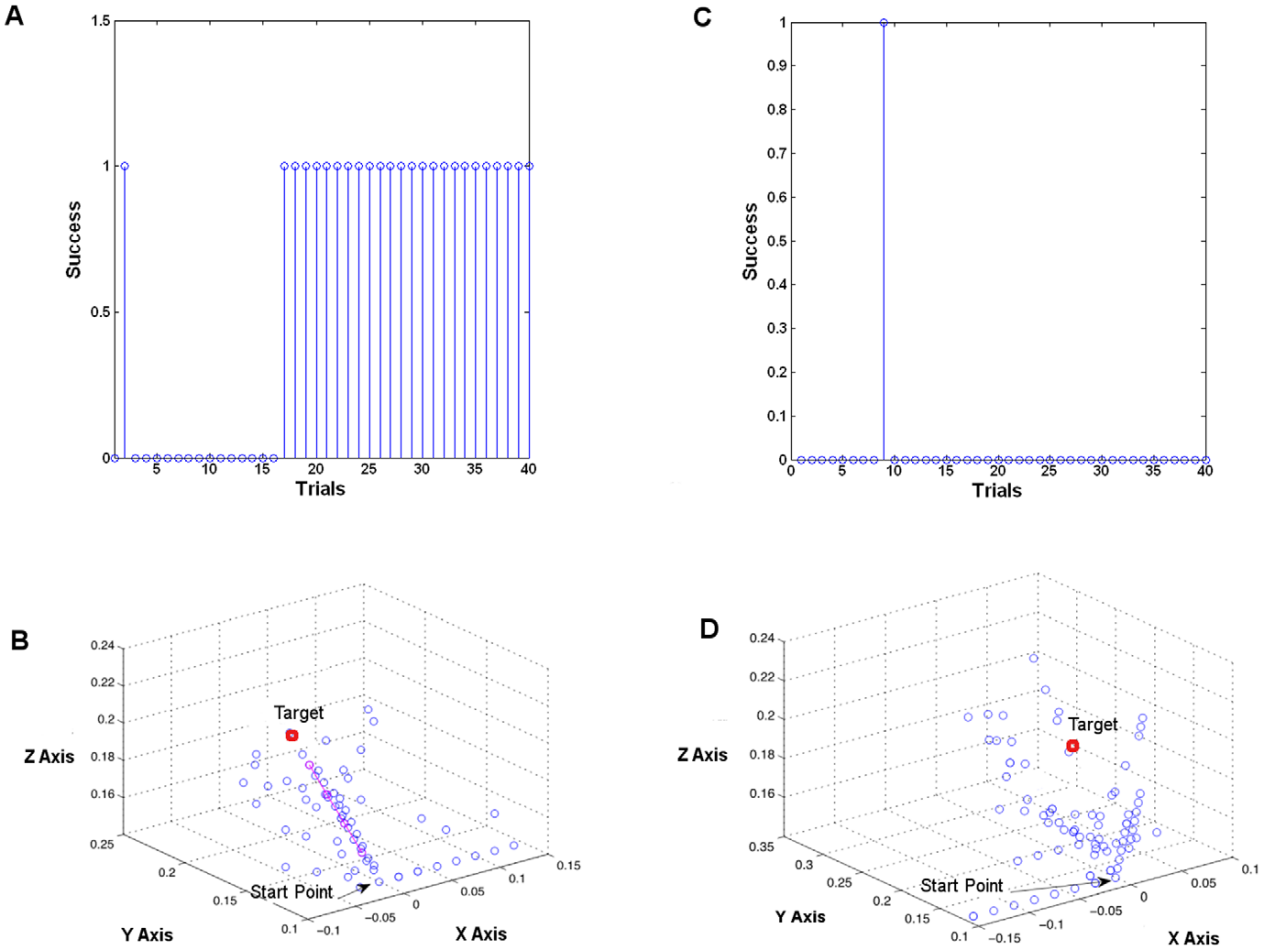
Actor-Critic decoding performance in navigating the robot to the target based on M1 neural activity. Target acquisition performance and robot trajectory in 3D space during adaptation of the Actor using: (A–B) an evaluative feedback extracted from the NAcc. (C–D) Random values as evaluative feedback.

Figure 9A shows the learning performance of the Actor during test trials (40 trials) based on M1 neural activity and Critic's evaluative feedback. Here it can be seen that after 16 trials the Actor converged to the solution and consistently navigated the robot to the target successfully. After this point, the performance was 100% for navigating to the target. Analysis of the trajectories formed by the Actor (see Figure 9B) indicated that early in the learning there was exploration of the space however, as the system converged to the solution the trajectory was focused on the direct path to the target. In order to show the effectiveness of the Critic's response based on the real NAcc neural activity, we performed a surrogate analysis in which the evaluative feedback from Critic was replaced by a random sequence of feedback values (+1 or −1). This surrogate analysis destroys the true evaluative feedback from the NAcc. If the Actor is relying on the evaluative feedback from the NAcc, then by altering its structure the Actor performance should degrade. In Figure 9C we can see the Actor was not able to solve the task with this random value since only one target was randomly acquired. In addition, the trajectories that were visited in 3D space were randomly distributed and did not follow the direct path to the target (see Figure 9D).

**Figure 10:**
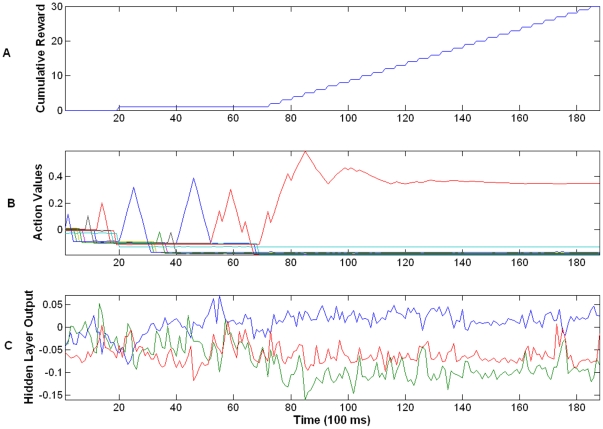
Actor's parameter adaptation during closed-loop control. (A) Cumulative reward over time. (B) Action values computed at the output layer of the Actor. Each color represents the value of a specific action. Here the red corresponds to the action that navigates the robot in a direct path to the target. (C) Output of the 3 hidden layer processing elements of the Actor. Larger adaptation of the values occurs before the “knee” of the cumulative reward curve. After the “knee” the system parameters stabilize their relative values indicating consolidation of the performance.

Analysis of the parameters of the Actor-Critic revealed interesting properties of the adaptation that indicated how the system was able to solve the task. Figure 10A shows the cumulative performance of the Actor-Critic architecture over time (concatenating the trials). In this figure it can be seen that the Actor initially had poor performance up to trial 15 but after this point performance rapidly increased. It was able to solve the task by increasing the value of the appropriate action needed to acquire the target. The red trace in Figure 10B corresponds to the action Forward-Right-Up that navigates the robot to the direct path to the target. Increasing the value of the appropriate action was a consequence of finding the right projection at the hidden layer weights (see Figure 10C). A powerful property of this architecture that can be seen in Figure 10C is that prior to the increase in performance there is large adaptation of the model parameters indicating the learning process. However, once the performance begins to increase the adaptation of these parameters reduces and they stabilize indicating a consolidation of the performance.

## Discussion

During daily life activities, BMIs should be able to perform well in complex tasks under conditions of dynamic environments and neuronal activation. In this paper, we developed a new framework to contend with these conditions in which goal-directed BMIs evolve with the user as an Intelligent Assistant through a value-based decision making process. The main research question in this regard was how two different entities (artificial and biologic) could engage in a symbiotic relationship. The key concept in promoting a symbiotic relation between the user and IA was to link the Perception-Action-Reward Cycles of the user and IA by sharing their goal. A challenge in this regard was to match the neural representation of goal in the brain with the mathematical definition of goal in the IA. We adopted an Actor-Critic method for the implementation of S-BMI because it was a goal-driven architecture that had separate structures for representation of goal (Critic) and action (Actor). Here, action selection in the Actor-Critic architecture was based on biologic goal information presented by Critic in the form of an evaluative feedback. Therefore, by extracting an evaluative feedback directly from the brain, the Actor could learn how to take action based on user's goals. We formulated the S-BMI control as a decision making process where the Actor learned action values in each neural state. Instead of a specific context dependent mapping, in a S-BMI, the Actor learned a control policy for associating neural states to actions. Goal-directed adaptation of the Actor played a pivotal role in aligning the control policy in the direction of the user's goal. For the control of neuroprosthesis, the evaluative feedback was used only for adaptation of the Actor structure when the user needed help (e.g. novel environment; otherwise, the S-BMI would not change the control policy).

Compared to other BMIs trained with an external teaching signal, the first step in the Actor-Critic design of S-BMI was to extract an internal measure of user's goal in the form of evaluative feedback from the brain. We investigated the possibility of extracting such a signal from NAcc for adaptation of the Actor. Our results suggested that NAcc contained rich representation of goal information during goal-approaching behavior. An important aspect of an evaluative feedback is that it has to contain both positive and negative reinforcement where the positive component predicts reward the negative component predicts aversion. We identified that bilateral selective neurons showed preference specific targets by decreasing their firing rate as the animal approached that target. These neurons were good candidates for extraction of evaluative feedback because they could predict both positive and negative reinforcement.

In a simulation study, we tested the adaptation of the Actor based on the NAcc evaluative feedback in two conditions; changing environments and in presence of dynamic neural states in M1. The Actor was able to adapt its control policy in changing environments to solve novel tasks. In all of our simulations, we observed that by changing the environment the Actor adapted its control policy accordingly to utilize actions that were required for solving the task. One of the appealing characteristics of S-BMI was that if a new task was within the space spanned by a learned control policy, the Actor was able to accomplish the task without need for adaptation. In other words, the Actor adapted its control policy only if it was not able to accomplish the task. In the S-BMI framework the Actor adapts to the user only if the performance degrades however, adaptation of the learning rate based on a measure of performance is the subject of future research. Adaptation of the control policy for novel tasks required utilizing new sequence of actions; however, in the case of changing neural patterns, the Actor needed to find a new mapping between the neural state and actions. We introduced a new neural pattern by shuffling the action preference of neurons. Again, the IA could associate the new neural state to appropriate actions just using an evaluative feedback.

Knowledge from the simulations was used to fine tune closed-loop S-BMI using real M1 and NAcc recordings. Compared to a surrogate analysis, the real NAcc evaluative feedback provided a useful method for adapting the Actor to solve a 3-D reaching task. One of the challenges we encountered was determining the scalar feedback needed to adapt the actor from the neural population information. Here, a standard MLP was used but more sophisticated methods in the future could be used to complete this task in either a supervised or unsupervised manner. In addition for real-time BMI control, evaluative feedback on single trial basis with high temporal resolution is desirable. This is one of the challenges in the Actor-Critic design of S-BMI because incorrect prediction of the NAcc neural activity can lead to inaccuracy in the Critic's response and degrade the Actor's performance. By increasing the information bandwidth of the evaluative feedback, more advanced signal processing techniques should be incorporated to increase the robustness of the value estimation. Another alternative to mitigate this problem is to decrease the sensitivity of the Actor to inaccuracy in the evaluative feedback. Based on the learning algorithm that was presented in this work the Actor was reasonably robust and able to converge to a solution based on 72% classification performance of the Critic. Our preliminary results also systematically showed how the S-BMI was robust to various noise properties in the evaluative feedback [50].

The Actor-Critic architecture gives IA great flexibility to adapt to both changes in the environment and the neural states. As far as there are repeatable sets of neural states that correlate with the task, the IA autonomously associates them to appropriate actions in such a way to maximize user's goals. Since the IA uses the brain's computational capability for reward/punishment prediction, the S-BMI is more computationally efficient than the conventional Actor-Critic method. However, we should consider the computation required for estimating the evaluative feedback from the neural ensemble activity in the brain. Adaptation of value estimator is the subject of our future research.
